# Patterns of Clinically Significant Symptoms of Depression Among Heavy Users of Alcohol and Cigarettes

**Published:** 2008-12-15

**Authors:** Joan Faith Epstein, Marta Induni, Tom Wilson

**Affiliations:** Survey Research Group Section, Cancer Surveillance and Research Branch, California Department of Public Health, 1700 Triute Rd, Sacramento, CA 95815-1402; California Department of Public Health, Sacramento, California; California Department of Mental Health, Sacramento, California

## Abstract

**Introduction:**

Depression is among the most prevalent and treatable diseases, and it is associated with cigarette smoking and heavy alcohol use. This study estimates the prevalence of depression, its variation among demographic subgroups, and its association with heavy alcohol use and cigarette smoking in California.

**Methods:**

The 2006 California Behavioral Risk Factor Surveillance System (BRFSS) includes the 8-item Patient Health Questionnaire, a standardized instrument used to measure depressive symptoms. We used findings from the 2006 BRFSS to calculate the prevalence of depression in California; we used logistic models to explore the relationships between depression, alcohol use, and smoking.

**Results:**

We found that 9.2% of adults in California had clinically significant depressive symptoms. Logistic models indicated that daily smokers were more than 3 times more likely to have clinically significant depressive symptoms than were nonsmokers, and heavy drinkers were approximately 3 times more likely to have clinically significant depressive symptoms than were nondrinkers.

**Conclusion:**

Because heavy alcohol use and daily smoking are each associated with depression, people who do both may be at an increased risk for depression. This is a public health issue because people who drink alcohol often also smoke and vice versa. Intervention efforts might target persons who are users of both these drugs, and practitioners should be aware that smokers who are heavy alcohol users are at an increased risk for depression.

## Introduction

Depression is a leading cause of disability worldwide (1) and is among the most prevalent and treatable diseases (2). Cigarette smoking and heavy alcohol use, which are closely linked (3), are associated with a number of physical illnesses, including cancer and cardiovascular, respiratory, and other chronic diseases (4-7), and both are associated with depression. According to the National Survey on Drug Use and Health, 7.2% of all US adults in 2006 had had at least 1 major depressive episode (MDE) in the previous year (8). Estimates from this study indicated a strong association between MDE and daily cigarette smoking and between MDE and heavy alcohol use. Among adults with a history of MDE, 8.6% were heavy alcohol users; among adults who reported no MDE, the rate was 7.3%. Similarly, among adults with a history of MDE, the rate of daily cigarette use was 29.7%, and among adults who reported no MDE, the rate was 16.0%. When associations between drinking and MDE were estimated from the 2000-2001 National Epidemiologic Survey on Alcohol and Related Conditions, they indicated that among heavy drinkers, 9.0% had had an MDE in the previous year, and among light drinkers the rate was 7.9%. The overall rate among adults in the United States was 7.1% (9).

Measuring mental illnesses in a population survey is problematic, not only because many people are not aware of their illness but also because diagnostic scales used by clinicians are generally too long or too cumbersome to be included in a general population survey. The Patient Health Questionnaire (PHQ-8) is a short, 8-item depression scale to diagnose depression and measure its severity (2). Because it is half the length of other depression scales, it is useful in population-based surveys.

Although estimates at the national level have shown an association between depression and alcohol use and between depression and cigarette smoking separately, we are not aware of any studies that have looked at the association between depression and heavy alcohol use among smokers or the association between depression and smoking among heavy alcohol users at the national and the state levels. We estimate the prevalence of clinically significant depressive symptoms in the California population and their variation among demographic subgroups. We also examine the association between clinically significant depressive symptoms and smoking and between clinically significant depressive symptoms and alcohol use. We assess the effect of smoking on depression while controlling for alcohol use and other confounders, the effect of alcohol use on depression while controlling for smoking and other confounders, and the combined effect of alcohol use and smoking on depression. We also demonstrate the use of the PHQ-8 in a general population survey in California.

## Methods

### Data source

We used data from the 2006 California Behavioral Risk Factor Surveillance System (BRFSS), an ongoing telephone survey of randomly selected adults that is designed to assess the prevalence of and trends in health-related behaviors in the California population aged 18 years and older. The 2006 BRFSS sample was randomly selected within 2 strata consisting of Los Angeles County and the rest of California. Interviewers made up to 16 calls at all times of the day to maximize the number of respondents. The final sample included 5,692 adults. The upper-bound response rate was 65% (the proportion of eligible households that completed the interview). This study was approved by the California Department of Public Health Committee for the Protection of Human Subjects.

### Dependent variable: depression symptoms

Having clinically significant depressive symptoms was the dependent variable for the logistic models in this study. Clinically significant depressive symptoms were measured by using the PHQ-8 (2). The 2006 BRFSS included the PHQ-8, a brief depression scale similar to the PHQ-9. The PHQ-9, a well-validated and widely used diagnostic and severity measure (10-12), consists of the 9 criteria on which the diagnosis of a major depressive disorder is based. Research suggests that it can be used without adjustment in diverse populations (13). Telephone administration of the PHQ-9 is also a reliable procedure for assessing depression in primary care (14). The only difference between the PHQ-8 and the PHQ-9 is that the PHQ-8 omits the 9th criterion ("thoughts that you would be better off dead or hurting yourself in some way") (2). In the BRFSS, each question asks about the number of days a symptom occurred during the last 2 weeks. To score the questions, days are converted to points (0-1 day = 0 points, 2-6 days = 1 point, 7-11 days = 2 points, and 12-14 days = 3 points) and summed to obtain a total score. The total score indicates the depressive symptom severity (a score of 1-4 indicates no to minimal depression, 5-9 indicates mild depression, 10-14 indicates moderate depression, 15-19 indicates moderately severe depression, and 20 or higher indicates severe depression). A person with a score of 10 or higher is defined as having clinically significant depressive symptoms. For this article, people who had clinically significant depressive symptoms in the previous 2 weeks were defined as having current depression.

### Independent variables

The main independent variables that could influence current depression were smoking and drinking. Other possible confounding variables that were independent variables in the model include age, race/ethnicity, sex, marital status, employment status, education level, body mass index (BMI), poverty status, vigorous exercise, and income.

Extent of drinking was classified into 4 categories: nondrinker (no alcohol use in the previous month), past-month drinker, binge drinker, and heavy drinker. For men, binge drinking was defined as having 5 or more drinks on at least 1 occasion during the preceding month. For women, binge drinking was defined as having 4 or more drinks on at least 1 occasion during the preceding month. Heavy drinking was defined as binge drinking on 5 or more occasions in the previous month. To make the categories mutually exclusive, past-month drinkers did not include binge drinkers, and binge drinkers did not include heavy drinkers.

Extent of smoking was also classified into 4 categories: nonsmoker, former smoker, current smoker, and daily smoker. Respondents were classified as current smokers if they reported having smoked 100 cigarettes or more during their lifetimes and acknowledged smoking 1 cigarette or more in the previous 30 days. Respondents were classified as former smokers if they had smoked 100 cigarettes or more during their lifetimes but had not smoked during the previous 30 days. Daily smokers reported smoking daily. To make categories mutually excusive, current smokers did not include daily smokers.

Employment status was coded as employed for wages, self-employed, out of work 1 year or more, out of work less than 1 year, homemaker, student, retired, or unable to work. Education level was based on highest grade of school completed and was coded as less than ninth grade, some high school, high school graduate or General Educational Development certified, some technical school, technical school graduate, some college, college graduate, or postgraduate. Self-reported weight and height were used to calculate BMI, and participants were classified into BMI categories according to Centers for Disease Control and Prevention guidelines (underweight, BMI <18.5 kg/m^2^; healthy weight, BMI 18.5-24.9 kg/m^2^; overweight, BMI 25.0-29.9 kg/m^2^; obese, BMI ≥30.0 kg/m^2^) (15). Participants were classified as participating in vigorous exercise if they reported doing activities such as running, aerobics, heavy yard work, or any other activity that caused increases in breathing or heart rate for at least 20 minutes on at least 3 days per week.

### Statistical analysis

To describe the magnitude of current depression in this population, we calculated rates of current depression by sociodemographic characteristics as well as other characteristics that might be related to depression. We used logistic regression to calculate odds ratios (ORs) for levels of alcohol and cigarette use. All estimates were weighted, and all standard errors were calculated by using SAS version 7.0 (SAS Institute Inc, Cary, North Carolina). All pairwise comparisons of estimates were tested for significance by using SUDAAN version 9.0.1 (RTI International, Research Triangle Park, North Carolina) to adjust for the complex sample design. We considered differences significant at *P* ≤ .05.

Logistic models were developed by using current depression as the dependent variable. Model 1 included drinking and smoking as the only dependent variables to determine the effect of smoking and drinking on depression, without controlling for other possible confounding and interacting variables. Model 2 included all possible confounders in BRFSS. In addition to smoking and drinking, this model included sex, race/ethnicity, age, marital status, education level, employment status, income, poverty status, BMI, and vigorous exercise.

Additional models were developed to determine the model with the most parsimonious fit by using Akaike's information criterion (AIC — in comparing 2 models, a lower AIC indicates more parsimonious fit) and to control for confounding and interacting variables. Adjusted odds ratios for smoking and drinking were estimated for each model, and their change from model to model was examined to determine the effect of confounding. An analysis of effects that indicated the significance of a variable in the presence of all other variables in the model was used to determine which variables to exclude from the model. Possible confounders that were not significant in the presence of other variables in the model were not included in the next model.

Model 3 included age, marital status, employment status, income, BMI, and vigorous exercise in addition to smoking and drinking. Each variable in this model was significant in the presence of all other variables in the model. Model 4 was the same as model 3 but included an interaction term for drinking and smoking. Model 4 had the most parsimonious fit with the lowest AIC value, but the ORs in this model were unstable.

## Results

### Descriptive analysis

We found a 9.2% prevalence of current depression in California, and prevalence was higher among Hispanics (12.1%) than among whites (7.2%). Current depression was most common in people aged 50 to 59 years (13.5%) and least common among people aged 60 or older (5.6%). The prevalence of current depression was lowest among married people (6.7%) and highest among divorced or separated people (17.9% and 17.4%, respectively). Rates of current depression generally decreased as annual household income rose. Rates were highest among people with the lowest income level (27.1% among people with an income <$10,000) and lowest among people with the highest income level (2.5% among people with an income >$100,000). People who did not participate in vigorous exercise were twice as likely to have current depression as people who participated in vigorous exercise (10.9% vs 5.0%). Obese people were more likely to be depressed than were those who were underweight, healthy, or overweight (16.5% vs 6.8%, 7.9%, and 5.8%, respectively).

Prevalence of current depression was higher among daily smokers (23.8%) and past-month smokers (22.5%) than among former smokers (11.2%) or nonsmokers (5.8%). Prevalence of current depression was highest among heavy drinkers (24.2%) and lowest among binge drinkers (7.8%) and past-month drinkers (6.0%). Among nondrinkers, the prevalence was 11.6%. Current depression was most common among daily smokers who were also binge drinkers (43.5%) or heavy drinkers (36.8%) and least common among people who never smoked and were past-month drinkers (3.6%) or binge drinkers (1.4%).

### Logistic regression models

In the model that did not account for possible confounding variables, smokers in all categories (daily, former, and current) were more likely than nonsmokers to have clinically significant depressive symptoms (Table 1). Heavy drinkers were somewhat more likely to have current depression than were nondrinkers, although binge and past-month drinkers were only approximately half as likely as nondrinkers to have current depression. The AIC measure of fit was highest for this model, which was expected because this model did not control for any possible confounding variables.

Model 2 (data not shown) included the most possible confounding variables available in BRFSS, and model 3 (Table 2) retained only those variables from model 2 that were significant in the presence of all other variables (race/ethnicity, sex, education level, and poverty status were dropped from model 2). In model 3, all levels of smoking (daily, former, and current) were associated with higher odds of depression than was nonsmoking, although the difference between former smokers and nonsmokers did not reach significance. Heavy drinkers were approximately 3 times more likely to report depression than were nondrinkers; binge and past-month drinkers were less likely to report depression than were nondrinkers, but the difference did not reach significance. These associations were nearly identical to those seen in model 2, which indicates that the eliminated variables were not true confounders.

Model 4 (Table 3) included the same variables as model 3 but also included an interaction term for smoking and drinking. The interaction between the smoking and drinking variables was significant, which indicates that the relationship between depression and smoking changes depending on an adult's drinking level, and the relationship between depression and drinking changes depending on an adult's smoking level. The odds of current depression for daily smokers compared with nonsmokers was more than 4 times higher among heavy drinkers than among nondrinkers (OR, 14.3 vs 2.9). Similarly, the odds of current depression for heavy drinkers compared with nondrinkers was more than 4 times higher among daily smokers than among nonsmokers (OR, 9.3 vs 1.9).

## Discussion

We found that smoking and heavy alcohol use are associated with current depression in California. Rates of current depression were more than 3 times as high among daily smokers and past-month smokers as among nonsmokers. Heavy drinkers were more than 3 times more likely to have current depression than were past-month drinkers and binge drinkers.

The association between depression and smoking and between depression and heavy drinking persisted even after controlling for confounding variables and examining interactions for smoking and alcohol use. In all models examined, the odds of depression were significantly higher for daily and past-month smokers than for nonsmokers. Similarly, in every model except the 1 that did not control for confounders, the odds of current depression were significantly higher for heavy drinkers than for nondrinkers.

The models with an interaction term and the descriptive statistics imply that heavy and binge drinking interact with smoking to increase the likelihood of current depression. People who both smoke and are heavy drinkers are more likely to have current depression than are those who do only 1 of these activities. Prevention efforts might target people who are dual users, and practitioners should be aware that smokers who are also heavy alcohol users are at an increased risk for current depression.

To the best of our knowledge, this is the first time the PHQ-8 has been included in a general population study in the United States and, therefore, the first time in a population-based study in California. We found a prevalence of current depression in California of 9.2%, which is consistent with the estimate of 9.2% seen in a validity study of the PHQ-9 that used a representative sample of the general population of Germany (16). Patterns of depression by most sociodemographic characteristics were similar to those seen in national, population-based surveys (17,18). We found no significant difference in prevalence by sex (8.7% in men and 10.1% in women), although national surveys indicate that depression is more prevalent in women (8).

Caution must be observed in interpreting these results. Because of the small sample sizes of some subgroups, estimates of ORs by different levels of the interacting variable have large standard errors, which indicates instability. Because this is a cross-sectional survey, these results do not indicate whether smoking and drinking cause depression or whether depression causes people to smoke and drink.

Another limitation of the study relates to the use of telephones. Because this study was conducted by telephone, it excludes people who do not have residential telephone service. Certain populations, such as very poor ones, may be missed by this type of survey (19), and the effect of this limitation on our findings is unclear. However, approximately 95% of US households are estimated to have at least 1 telephone (20), and approximately 92% of US adults live in households with landline telephone service (21). Telephone surveys with low response rates might not contain differential response bias compared with those with higher response rates and may increase reporting of sensitive behaviors compared with face-to-face surveys (22). In a study that evaluated the agreement between a self-administered and a telephone-administered PHQ-9, the intra-class correlation coefficient and weighted κ indicated excellent agreement between the administration procedures (14).

This study demonstrated the use of the PHQ-8 in a population-based survey. Because the PHQ-8 is half the length of many other depression scales and has comparable sensitivity and specificity, it may be useful in other studies of depression in which a longer instrument would not be feasible.

People who drink heavily tend to smoke heavily, and people who are dependent on alcohol are likely to be dependent on cigarettes (23). Over time, dependence on these drugs may lead to long-term changes in neuronal activity (24). Depression can complicate co-occurring alcohol and nicotine dependence and impede attempts to quit (25). Intervention efforts should target people who are dual users, and practitioners should be aware that smokers who are heavy alcohol users are at an increased risk for depression.

## Figures and Tables

**Table 1:** Logistic Regression Coefficients and Fit Statistics for a Model (Model 1^a^) That Examines the Relationship Between Current Depression and Smoking and Alcohol Use Among Adults Aged 18 or Older, 2006 California Behavioral Risk Factor Surveillance System

**Variable**	**β**	**SE of β**	***P* Value**	**OR (95% CI)**	**Type 3 Analysis of Effects**	

					**χ^2^**	***P* Value**
**Intercept**	−2.57	0.23	<.001	NA	NA	NA
**Extent of smoking^b^**						
Nonsmoker	1 [Reference]				34.6	<.001
Daily	1.76	0.37	<.001	5.82 (2.80-12.09)		
Former	0.69	0.31	.02	2.00 (1.10-3.63)		
Current	1.98	0.48	<.001	7.24 (2.82-8.60)		
**Extent of alcohol use^c^**						
Nondrinker	1 [Reference]				9.7	.02
Heavy	0.46	0.39	.23	1.59 (0.75-3.39)		
Binge	−0.55	0.53	.29	0.58 (0.21-1.61)		
Past month	−0.72	0.29	.01	0.49 (0.28-0.86)		

Abbreviations: SE, standard error; OR, odds ratio; CI, confidence interval; NA, not applicable.

a Akaike's information criterion: 9,805,316.

b Daily smokers reported smoking daily; former smokers reported smoking ≥100 cigarettes during their lifetimes but had not smoked in the previous 30 days; current smokers reported smoking ≥100 cigarettes during their lifetimes and ≥1 cigarette during the previous 30 days. Current smokers did not include daily smokers.

c Nondrinkers reported no alcohol use in the previous month; heavy drinkers reported binge drinking on ≥5 occasions in the previous month; binge drinkers reported having ≥5 drinks on ≥1 occasion (for men) or ≥4 drinks on ≥1 occasion (for women) in the previous month; past-month drinkers reported having ≥1 drink in the previous month. Past-month drinkers did not include binge drinkers, and binge drinkers did not include heavy drinkers.

**Table 2:** Logistic Regression Coefficients and Fit Statistics for a Model (Model 3^a^) That Examines the Relationship Between Current Depression and Smoking and Alcohol Use, Including Only Significant Confounders, Among Adults Aged 18 or Older, 2006 California Behavioral Risk Factor Surveillance System

**Variable**	**β**	**SE of β**	***P* Value**	**OR (95% CI)**	**Type 3 Analysis of Effects**	

					**χ^2^**	***P* Value**
**Intercept**	−6.09	0.66	<.001	NA	NA	NA
**Extent of smoking^b^**						
Nonsmoker	1 [Reference]				14.9	<.01
Daily	1.32	0.29	<.001	3.74 (2.11-6.65)		
Former	0.53	0.32	.09	1.70 (0.91-3.16)		
Current	1.04	0.43	.01	2.84 (1.23-6.55)		
**Extent of drinking^c^**						
Nondrinker	1 [Reference]				21.3	<.001
Heavy	1.07	0.45	.02	2.90 (1.21-6.95)		
Binge	−0.50	0.36	.17	0.61 (0.30-1.23)		
Past month	−0.28	0.28	.31	0.77 (0.44-1.30)		
**Age, y**						
18-24	2.61	0.63	<.001	13.59 (3.92-48.75)	18.9	<.01
25-34	1.94	0.56	<.001	6.96 (2.32-20.90)		
35-44	1.54	0.55	<.01	4.69 (1.61-13.66)		
45-54	1.42	0.56	.01	4.15 (1.38-12.45)		
55-64	0.92	0.56	.10	2.52 (0.84-7.60)		
≥65	1 [Reference]					
**Employment status**						
Employed for wages	1 [Reference]				87.1	<.001
Out of work <1 year	0.40	0.52	.60	1.49 (0.54-4.09)		
Out of work >1 year	1.20	0.60	.05	3.31 (1.02-10.68)		
Retired	0.79	0.52	.13	2.21 (0.79-6.17)		
Self-employed	0.32	0.38	.39	1.38 (0.66-2.89)		
Homemaker	0.06	0.41	.88	1.06 (0.48-2.36)		
Student	−0.51	0.64	.42	0.60 (0.17-2.10)		
Unable to work	3.31	0.37	<.001	27.26 (13.12-56.65)		
**BMI**						
≥30.0 kg/m^2^ (obese)	0.70	0.28	.01	2.01 (1.17-3.46)	19.9	<.001
25.0-29.9 kg/m^2^ (overweight)	−0.48	0.27	.08	0.62 (0.36-1.06)		
18.5-24.9 kg/m^2^ (healthy weight)	1 [Reference]					
<18.5 kg/m^2^ (underweight)	−0.59	0.48	.22	0.56 (0.22-1.41)		
**Annual income, $**						
<10,000	0.17	0.74	.82	2.63 (0.92-7.51)	21.9	<.01
10,000-14,999	−0.58	0.74	.44	1.22 (0.40-3.69)		
15,000-19,999	−0.81	0.78	.30	0.88 (0.28-2.78)		
20,000-24,999	0.53	0.71	.45	3.41 (1.29-9.03)		
25,000-34,999	0.65	0.61	.28	4.06 (1.46-11.33)		
35,000-49,999	0.75	0.50	.14	2.90 (1.08-7.77)		
50,000-74,999	0.53	0.44	.23	1.90 (0.79-4.58)		
75,000-100,000	0.16	0.50	.75	1.25 (0.46-3.38)		
>100,000	1 [Reference]					
**Marital status**						
Married	1 [Reference]				21.8	.001
Divorced	1.25	0.39	<.001	3.50 (1.62-7.55)		
Never married	0.07	0.30	.82	1.07 (0.60-1.91)		
Widowed	1.47	0.52	<.01	4.34 (1.56-11.05)		
Unmarried couple	0.97	0.40	.02	2.63 (1.20-5.76)		
Separated	0.59	0.51	.25	1.80 (0.67-4.83)		
**Vigorous exercise**						
Yes	0.40	0.13	<.01	2.20 (1.35-3.60)	9.9	<.01
No	1 [Reference]					

Abbreviations: SE, standard error; OR, odds ratio; CI, confidence interval; NA, not applicable; BMI, body mass index.

a Akaike's information criterion: 7,278,444.

b Daily smokers reported smoking daily; former smokers reported smoking ≥100 cigarettes during their lifetimes but had not smoked in the previous 30 days; current smokers reported smoking ≥100 cigarettes during their lifetimes and ≥1 cigarette during the previous 30 days. Current smokers did not include daily smokers.

c Nondrinkers reported no alcohol use in the previous month; heavy drinkers reported binge drinking on ≥5 occasions in the previous month; binge drinkers reported having ≥5 drinks on ≥1 occasion (for men) or ≥4 drinks on ≥1 occasion (for women) in the previous month; past-month drinkers reported having ≥1 drink in the previous month. Past-month drinkers did not include binge drinkers, and binge drinkers did not include heavy drinkers.

**Table 3:** Odds of Current Depression (Model 4^a^), Taking Into Account the Interaction Term for Smoking by Different Levels of Drinking, 2006 California Behavioral Risk Factor Surveillance System

**Extent of Smoking or Drinking^b^**	**OR**	**SE**	**95% CI**
**Extent of smoking among nondrinkers**			
Nonsmoker	1 [Reference]		
Daily	2.89	1.32	1.18-7.07
Former	1.65	0.72	0.70-3.86
Current	1.70	0.93	0.58-4.99
**Extent of smoking among past-month drinkers**			
Nonsmoker	1 [Reference]		
Daily	1.05	0.53	0.39-2.85
Former	1.55	0.70	0.64-3.74
Current	4.79	2.93	1.44-15.91
**Extent of smoking among binge drinkers**			
Nonsmoker	1 [Reference]		
Daily	39.50	32.1	8.04-194.00
Former	8.97	8.30	1.46-55.02
Current	5.04	6.03	0.48-52.56
**Extent of smoking among heavy drinkers**			
Nonsmoker	1 [Reference]		
Daily	14.30	14.43	1.98-103.30
Former	0.24	0.34	0.01-4.14
Current	4.56	5.23	0.48-43.15
**Extent of drinking among nonsmokers**			
Nondrinker	1 [Reference]		
Heavy	1.87	1.58	0.36-9.78
Binge	0.16	0.11	0.04-0.62
Past month	0.79	0.33	0.35-1.78
**Extent of drinking among former smokers**			
Nondrinker	1 [Reference]		
Heavy	1.87	1.58	0.36-9.78
Binge	0.85	0.63	0.20-3.63
Past month	0.79	0.33	0.35-1.78
**Extent of drinking among current smokers**			
Nondrinker	1 [Reference]		
Heavy	5.02	4.92	0.74-34.20
Binge	0.46	0.50	0.06-3.86
Past month	2.23	1.60	0.55-9.09
**Extent of drinking among daily smokers**			
Nondrinker	1 [Reference]		
Heavy	9.26	6.89	2.16-39.79
Binge	2.13	1.33	0.62-7.25
Past month	0.29	0.16	0.10-0.84

Abbreviations: OR, odds ratio; SE, standard error; CI, confidence interval.

a Akaike's information criterion: 7,091,514.

b Daily smokers reported smoking daily; former smokers reported smoking ≥100 cigarettes during their lifetimes but had not smoked in the previous 30 days; current smokers reported smoking ≥100 cigarettes during their lifetimes and ≥1 cigarette during the previous 30 days. Current smokers did not include daily smokers. Nondrinkers reported no alcohol use in the previous month; heavy drinkers reported binge drinking on ≥5 occasions in the previous month; binge drinkers reported having ≥5 drinks on ≥1 occasion (for men) or ≥4 drinks on ≥1 occasion (for women) in the previous month; past-month drinkers reported having ≥1 drink in the previous month. Past-month drinkers did not include binge drinkers, and binge drinkers did not include heavy drinkers.
